# Dispersal promotes stability and persistence of exploited yeast mutualisms

**DOI:** 10.1093/ismejo/wraf003

**Published:** 2025-01-09

**Authors:** Cong Liu, Mayra C Vidal

**Affiliations:** Biology Department, University of Massachusetts Boston, Boston, MA 02125, United States; Museum of Comparative Zoology, Harvard University, Cambridge, MA 02138, United States; Biology Department, University of Massachusetts Boston, Boston, MA 02125, United States

**Keywords:** biodiversity, community stability, dispersal, experimental ecology, microbe, mutualism

## Abstract

Multispecies mutualistic interactions are ubiquitous and essential in nature, yet they face several threats, many of which have been exacerbated in the Anthropocene era. Understanding the factors that drive the stability and persistence of mutualism has become increasingly important in light of global change. Although dispersal is widely recognized as a crucial spatially explicit process in maintaining biodiversity and community structure, knowledge about how the dispersal of mutualists contributes to the persistence of mutualistic systems remains limited. In this study, we used a synthetic mutualism formed by genetically modified budding yeast to investigate the effect of dispersal on the persistence and stability of mutualisms under exploitation. We found that dispersal increased the persistence of exploited mutualisms by 80% compared to the isolated systems. Furthermore, our results showed that dispersal increased local diversity, decreased beta diversity among local communities, and stabilized community structure at the regional scale. Our results indicate that dispersal can allow mutualisms to persist in meta-communities by reintroducing species that are locally competitively excluded by exploiters. With limited dispersal, e.g. due to increased fragmentation of meta-communities, mutualisms might be more prone to breakdown. Taken together, our results highlight the critical role of dispersal in facilitating the persistence of mutualism.

## Introduction

Mutualistic interactions are ubiquitous in nature, playing critical roles in maintaining biodiversity and offering essential ecosystem services such as pollination, seed dispersal, and nutrient cycling [1–4]. Despite their prevalence and importance, mutualistic systems face several threats exacerbated in the Anthropocene, such as involving erosion of benefits, mismatches in space or time, loss of species, and reduction of movement between communities [5, 6], potentially leading to the breakdown of mutualistic interactions [3]. Breakdown of mutualisms could have cascading effects on the entire community, as primary productivity often relies on mutualistic interactions. Therefore, understanding the ecological dynamics of mutualism, as well as what facilitates the stability and persistence of mutualistic systems, is crucial for the effective management and conservation of ecosystems, particularly in light of the accelerating biodiversity loss under global change [7, 8].

Previous theoretical and experimental studies on the dynamics of mutualistic communities have identified several key factors and processes contributing to the stability and persistence of mutualism [4, 9, 10]. For example, species richness and the number of interacting partners have been associated with mutualism stability and persistence [11]. Redundancy and richness of mutualistic partners have also been shown to facilitate mutualism persistence, particularly in exploited mutualisms when mutualistic communities are challenged by the presence of exploiters or cheaters, who take advantage of commodities exchanged in mutualism without offering rewards [4]. However, cheaters can potentially reduce species richness by competitively excluding mutualist species [4]. Although theoretical studies have predicted the effect of processes operating at multiple spatial scales on the persistence of mutualisms [12–15], empirical evidence testing these predictions remain limited, particularly in experimental systems.

One crucial spatial explicit process essential in maintaining biodiversity, community structure, and ecosystem functions is dispersal between communities. Dispersal’s impact on species diversity and community stability varies across spatial scales [16–20]. At the local scale, dispersal is vital for preventing local extinctions by allowing species from favorable habitats (“source”) to recolonize less favorable habitats (“sink”), thereby promoting local species coexistence and increasing species diversity [16, 21–24]. At the regional scale, dispersal can help maintain a stable species pool by reintroducing species into communities where they are locally excluded, promoting the coexistence of all the local species, and contributing to overall community stability [7, 17, 25, 26]. Given these effects of dispersal on community structure across scales, dispersal could prevent the local extinction of mutualists and stabilize the mutualistic communities. This is especially valuable for exploited mutualisms, as mutualists tend to face higher local extinction rates due to the presence of cheaters that have higher competitive advantage due to lack of costs associated with the mutualism [3, 27–29]. However, only a limited number of theoretical studies have investigated the effect of dispersal on the dynamics of mutualistic interactions [30, 31], and none considered the potential for dispersal to facilitate the persistence of exploited mutualisms. Empirical examinations of the potential role dispersal has on the persistence and stability of mutualisms are of central importance to biodiversity conservation, particularly in the current context where local diversity and the flow of species between communities are being altered by global change.

Dispersal processes are often constrained due to anthropogenic disturbance such as habitat fragmentation, which limits the movement of species across landscape [32]. Conservation efforts increasingly address these limitations through strategies like directed dispersal to help restore populations facing decline and extirpation [33]. Directed dispersal involves intentionally moving individuals from populations with high density and genetic diversity to areas where populations are at risk, similar to conservation interventions aimed at sustaining biodiversity in fragmented environments. Although directed dispersal is selective and nonrandom, it mimics certain natural ecological scenarios, particularly in fragmented landscapes, where dispersal is often nonrandom and directed by population density, individuals with traits that facilitate dispersal, or habitat quality [34]. In this study, we specifically employed a directed dispersal approach to assess the effect of dispersal on the persistence and stability of mutualisms, reflecting conservation practices designed to prevent mutualism breakdown. Using directed dispersal in our experiment allowed us to precisely investigate how mutualist reintroduction could buffer against the destabilizing effects of cheaters. Although this approach is more controlled, it offered a practical and ecologically relevant way to study the dynamics of mutualistic persistence in our experimental system.

To test the effect of dispersal from source to sink populations on the persistence and stability of exploited yeast mutualisms, we directionally dispersed mutualists and cheater among local communities using a laboratory experiment based on a synthetic *Saccharomyces cerevisiae* mutualism [4]. In this system, yeast strains are genetically different and reproductively isolated, thus ecologically representing species with substantial niche overlap and increased potential for competitive exclusion. We hypothesize that the dispersal of mutualists will enhance persistence and reduce the collapse of mutualistic interactions within local yeast communities. Specifically, we predict dispersal of mutualists will prevent species loss through the rescue effect especially in exploited mutualisms, which could facilitate the persistence of mutualistic communities despite the negative exploitation impacts. Moreover, we expect that dispersal will homogenize species assemblages across communities and reduce beta diversity, thereby stabilizing the community structure at the regional scale.

## Material and methods

### Yeast mutualism system

**Figure 1:**
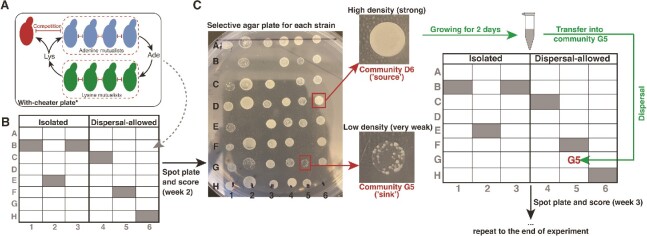
Scheme of the experiment setup. (A) Yeast species composition. Mutualist-only communities contain four strains of adenine mutualist (top) that release adenine (Ade) into the medium, which is taken up by the lysine mutualists (bottom) that release lysine (Lys), which is used by the adenine mutualists. With-cheater communities have a lysine cheater (top-left) that competes with the adenine mutualists for lysine. Blunt-ended arrows represent competition between strains. (B) Each community type, either exploited or mutualist only, was set up on the 48-well deep-well plates, with six blank wells (represented by the filled wells) per plate for contamination monitoring. Each plate contained only one community type; half of the plates were set up as isolated communities, and half were dispersal-allowed communities. (C) Scheme of dispersal. Each selective plate had a specific combination of amino acids or antibiotics that only allowed one strain to grow. In communities where dispersal was allowed, we reintroduced each strain when it was going extinct (<20 single colonies) from a community where the same strain was growing strongly.

We used genetically modified brewer’s yeast (*S. cerevisiae*) strains originally created from previously published studies [4, 35] to develop synthetic mutualistic communities. These communities included two types of mutualists, each overproducing and releasing a specific nutrient essential for yeast survival and growth but incapable of producing the other. The first type, adenine mutualists (AdeOPs), overproduces adenine, which is essential for yeast cell division but cannot produce lysine. The second type, lysine mutualists (LysOPs), overproduces lysine, which is important for yeast cell growth but cannot produce adenine (Fig. 1A, Table S1). Hence, when together, AdeOPs and LysOPs form a nutritional mutualism where the AdeOPs consume lysine produced by LysOPs, and LysOPs consume adenine produced by AdeOPs. In addition, we included a “cheater” strain that consumes lysine without contributing any resources (“lysine cheater”) in the communities. All strains are asexual and were modified to exhibit minor variations in both genotype and phenotype (Table S1), making them analogous to closely related species with similar ecological roles. This leads to intense competition at the local scale (local communities) due to the significant overlap in their ecological niches. The cheater also has a competitive advantage over the AdeOPs, as they compete for the same resource (lysine), but the mutualist bears a fitness cost of overproducing adenine [4]. All mutualist strains grew in a liquid medium lacking adenine and lysine, making their survival and reproduction dependent on the presence of at least one strain of each mutualist type, thus forming an obligate mutualism. Similarly, cheaters cannot survive independently in this media, and it requires the presence of mutualists who produce lysine (LysOP). However, when the cheater competitively excludes all AdeOPs, the community eventually collapses as the LysOPs cannot survive without adenine provided by the AdeOPs. Thus, the persistence of these mutualistic communities hinges crucially on having both types of mutualists present, and the competitive exclusion of all strains of AdeOPs by the cheater consequently leads to community extinction.

To set up the experiment, we created mutualist-only communities that contained eight mutualist strains and exploited mutualistic communities that included eight mutualists and one cheater strain (Fig. 1A and B, Table S1). Of the mutualist strains, four were AdeOPs and four were LysOPs. We used the same combination of strains as described in previous study [4], in which the strains represent different combinations of removal of the ability to produce histidine, leucine, uracile, tryptophan, or the addition of hygromycin resistance (see the complete list of strain genotypes and corresponding selective plates in Supplementary Materials Table S1). The combination of genetic modifications makes it possible to tell the strains apart in selective agar plates and introduce phenotypic differences (e.g. differences in growth and starvation resistance), which influences the competitive landscape among strains [4].

### Experimental design

Yeast strains were grown overnight in liquid YPD (yeast extract peptone dextrose media composed of 1% [w/v] yeast extract, 2% [w/v] peptone, and 2% [w/v] dextrose), and then washed in sterile water before being used to assemble the experimental communities at the same total starting density of 0.1 OD_600_. For mutualist-only communities, we set the starting ratio of adenine overproducers (AdeOP) and lysine overproducers (LysOP) to 1:1 by adding 0.0125 OD_600_ of each of the eight mutualist strains. In contrast, we used the 2:2:1 starting radio for AdeOP: LysOP: cheater to assemble the exploited mutualistic communities by adding 0.01 OD_600_ of each of the eight mutualist strains and 0.02 OD_600_ of the cheater. This ratio of 2:2:1 assumes a higher density of mutualist guild compared to cheater, as is expected to be found in natural systems. Each community type, either exploited or mutualist only, was set up in two separate 48-well deep-well plates, with six blank wells per plate for contamination monitoring (Fig. 1B). This setup resulted in a total of 168 communities, split equally between exploited (two plates, *n* = 84) and mutualist-only (two plates, *n* = 84) types. Each plate contained only one community type, meaning that one plate was entirely mutualist-only and the other entirely exploited. To test the effect of dispersal on the persistence of mutualisms, we allowed dispersal in half of the communities in each plate (“dispersal-allowed communities,” Fig. 1B), whereas the remaining half were kept isolated (“isolated communities,” Fig. 1B). Thus, within each plate, there were 21 isolated communities and 21 dispersal-allowed communities.

Communities were grown in 2 ml the synthetic dextrose (SD) medium lacking adenine and lysine at 30°C and 50%–70% humidity on a rotating wheel. We transferred 200 μl of each culture into 1800 μl of fresh medium every 48 h for a total experiment period of 8 weeks. At the end of each week, we examined the presence and abundance of each strain in each community by plating 10 μl the communities on selective agar plates (Fig. 1C). We qualitatively estimated the density of strains by considering the completeness of the dot formed by grown yeast in the selective agar plates. If the dot was completely covered with yeast (“strong”), this indicated a high density of the strain in the original liquid media. If the spot had an uneven border (“weak”), it indicated a slightly lower density, whereas the presence of distinct, countable colonies or visually apparent small distinct colonies signified populations at low densities in the trajectory to extinction (“very weak” or “micro,” respectively). The absence of yeast showed that the strain was locally extinct (“gone”; more details of the agar plate scoring can be found in the supplementary materials Fig. S1).

Directional dispersal was applied for all strains, including the cheater, in the dispersal-allowed communities when the strain showed “very weak” scoring with less than 20 individual colonies (Fig. 1C). For each dispersal-allowed community, we first identified the “sink” population, where a strain was very weak, and the “source” communities, where the same strain was strong. The source strains were always selected from other dispersal-allowed communities within the same plate. We then obtained the “source” population strain from selective agar plates, and grew it in liquid YPD for 2 days, and followed the washing procedure before adding it to the “sink” communities. Hence, we only introduced individual strains from source communities where the population of the specific strain was at high density, to sink communities, where the population of the specific strain was at very low density, nearing extinction. The amount of strain dispersed to the community was set to be 10% of the average strain density in the local community right after transferring to simulate the natural dispersal processes [36]. We let the strains established and checked their persistence after two days by platting the community in selective agar plates. The entire experiment consisted of 653 dispersal events (more details can be found in Fig. S2). The directional dispersal method in this study was designed to mirror conservation approaches where individuals from populations with high genetic diversity are introduced to threatened populations with the goal of reversing local extinction [37, 38]. This approach reflects nonrandom density-dependent dispersal in nature, where species often disperse from robust populations to recolonize areas where they are locally declining or extirpated, thereby enhancing persistence and stability across the meta-community [39]. Thus, we cannot separate the effect of dispersal from selective replenishment of sink populations.

### Investigation of factors associated with strain abundance

Before performing the directional dispersal, we investigate the importance of several predictors (type of community, type of mutualist, and yeast strains) in determining the strain abundance (measured based on the abundance of each strain in selective agar plates) at Week 2, when abundance started to vary across communities. This test allowed us to consider potential differences in the competitive ability of strains before we applied the dispersal treatments. We focused on Week 2 because this was the time-point when we started to observe significant reduction in the density of strains that required rescue by dispersal; before then, all strains had high (“strong”) or medium (“weak”) densities. For this statistical analysis, we fitted generalized linear mixed models (GLMMs) with the Bayesian Markov chain Monte Carlo method using the “MCMCglmm” package Version 2.35 [40]. We first quantified the importance of the community type (mutualist only vs. exploited) and mutualist type (AdeOP vs. LysOP) on the strain abundance by including those variables as fixed effects, the community ID and strain species were the random effects (“model a”). We then tested whether different strain species of the same mutualist type affect the strain abundance by running two separate models on AdeOPs (“model b”) and LysOPs (“model c”) with community ID as a random effect.

### The effect of dispersal on alpha diversity

To test whether dispersal prevents species loss of mutualistic communities, we calculated the alpha diversity of yeast communities based on Shannon’s diversity using the R package ``hillR'' Version 0.5.2 [41, 42]. Shannon’s diversity was measured by counting the species weighted in proportion to their relative abundance, representing the effective number of common species in the community. The abundance of each yeast strain was estimated based on its density on the selective agar plate every week, but we focus on the effect of dispersal on alpha diversity at the end of the experiment (Week 7), as the community dynamics greatly varied from week to week due to species introductions.

We used MCMCglmm to investigate the effect of dispersal on alpha diversity. In this case, dispersal (categorical variable) is the fixed effect and plate ID as well as community ID was treated as the random effect. For each model, we used a Gelman-prior [43] for the fixed effect (B) and list G = (G1 = (V = diag(1), nu = 1) priors for the random effect. We ran each model for 240 000 iterations, with a burn-in period of 30 000 and a thinning interval of 100, to obtain >1000 posterior samples of each chain for parameter estimation. Model convergence and chain mixing were assessed by examining the trace plots after each run. For each model, the mean and 95% credible intervals (95% CI) of parameter estimates from the posterior distributions were reported, and the significance of the estimate was considered if the 95% CI for the coefficient did not overlay with zero.

To assess whether there was a significant difference in the rescue effect of dispersal, we measured the abundance of each strain after dispersal and calculated the proportion of recovered strains as the strains that reached high density (“strong”) when plated 2 days after reintroduction. We then used GLMMs to analyze the effects of community type (exploited vs. mutualist only) on the proportion of recovered strains. In our GLMM, the response variable was the proportion of recovered strains with community type as a fixed effect. The dispersal-allowed communities within the same plate may not be fully independent due to potential dispersal among them. To account for this potential pseudo-replication, we included plate ID as a random effect in all analyses, along with community ID as a random effect to capture community-specific variation. This approach controls for any shared influences within plates, ensuring accurate estimates of treatment effects. The GLMM was conducted using the R package glmmTMB version 1.1.10 [44].

### The effect of dispersal on beta diversity

To test if dispersal decreases beta diversity among local communities and stabilizes community structure, we calculated the beta diversity of all mutualist-only communities for both dispersal-allowed and isolated treatments using the abundance-based Bray–Curtis dissimilarity index with R package “*betapart*” version 1.6 [45]. We did not calculate the beta diversity among the exploited communities due to the fact that most of the species went locally extinct after 3 weeks when cheaters were present in the isolated communities (Fig. S3). To visualize the effect of dispersal on the yeast beta diversity and community structure, we performed a 2D nonparametric multidimensional scaling (NMDS) based on the value of beta diversity between communities using the R package *vegan* Version 2.6–4 [46].

To further explore the underlying processes that shaped mutualistic community structure, we used the null model method by comparing the observed beta diversity to those expected under a null model. Dissimilarity of two communities significantly higher or lower than expected by chance indicates a deterministic community assembly (e.g. biotic interactions or environmental filtering, e.g. when there is a specific strain that always outcompetes others), whereas nonsignificant deviations from the null model suggest a stochastic assembly (e.g. random species loss). We used the standardized effect sizes to compare the observed and expected beta diversity. We first generated 1000 random communities by randomizing the community data matrix while keeping the species richness. We then calculated the standardized effect sizes of pairwise beta diversity (SES.TBD) using the formula below, where Mean_obs_ is the mean of observed beta diversity; Mean_null_ and s.d._null_ are the mean and standard deviance of beta diversity across all 1000 randomized communities.


$$ \text{SES} = (\text{Mean}_{\text{obs}}- \text{Mean}_{\text{null}})/\text{s.d.}_{\text{null}} $$

We used GLMMs to examine the changes in beta diversity over time. For dispersal-allowed and isolated communities, we regressed the response variable (beta diversity) against time (weeks). Because the beta diversity of any pairwise communities is not completed independent across different weeks, we gave the unique ID for each pairwise comparison and used it as a random effect in the model. Additionally, we include plate ID as random effect. We used Welch’s two-sample *t*-test to investigate whether the effect size of pairwise beta diversity is significantly different from the null expectation. All the analyses were performed in R version 4.3.1.

## Results

### Stochastic species loss within local mutualistic communities

When investigating the importance of the type of community (exploited vs. mutualists only), the type of mutualist (LysOP vs. AdeOP), and yeast genotypes in determining the strain abundance in Week 2 (before dispersal), we found that strain abundance in exploited communities was significantly lower than in mutualist-only communities, with a mean 30% reduction in abundance (*P*_MCMC_ < .001, ESS = 2100; Table 1, Model a). Moreover, strains of the LysOP mutualist type were 1.24 times more abundant than the AdeOPs (*P*_MCMC_ < .001, ESS = 2100; Table 1, Model a). However, strain genotypes did not significantly influence abundance when only considering the same mutualist type (Table 1, ESS = 2100, Models b and c), indicating random changes in abundance of the same mutualist types in the local communities. Hence, there was not a specific strain more likely to go extinct or to outcompete than others.

**Table 1:** Summary of MCMCglmm models (a, b, or c) predicting the strain abundance by the type of mutualism, the type of mutualist, or yeast strains.

**Model**	**Posterior mean**	**95% CI**	**ESS**	***P***_**MCMC**_
**(a) Overall**				
*Mutualism type (exploited)*	−0.160	[−0.268, −0.058]	2100	.004^**^
*Mutualist type (LysOP)*	0.189	[0.057, 0.318]	2100	.01^*^
**(b) AdeOP only**				
AdeOP2	−0.183	[−0.388, 0.027]	2100	.08667
AdeOP3	−0.087	[−0.291, 0.129]	2100	.42000
AdeOP4	−0.165	[−0.364, 0.061]	2100	.11810
**(c) LysOP only**				
LysOP2	−0.01578	[−0.230, 0.175]	2100	.1238
LysOP3	−0.01021	[−0.230, 0.192]	2100	.4381
LysOP4	−0.02885	[−0.221, 0.193]	2100	.2861

All the predictors are categorical variables between two (e.g. mutualism type and mutualist type in model a) and four levels (four AdeOP strains and four LysOP strains in model b, and c, respectively, represented by the numbers 1–4, with AdeOP1 or LysOP1 being in the intercept). Significant predictors are highlighted in italics, and the significant P-values are bolded. Asterisks represent *P*<0.05 (^*^) or *P* < 0.01 (^**^). Abbreviations: 95% CI, 95% of credible intervals; ESS, effective sample size. *P*_MCMC_, *P*-values from the Bayesian GLMMs.

### Dispersal increases alpha diversity and prevents species loss and community breakdown

**Figure 2:**
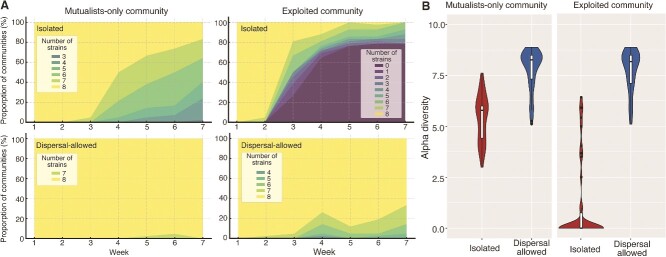
Dispersal prevents species loss and community breakdown in the multispecies mutualism system. (A) Proportion of communities retaining difference number of strains over time; (B) alpha diversity based on Hill number *q* = 1 by the end of experiment (Week 7) for mutualist-only and exploited communities.

Although all strains in both mutualist-only and exploited mutualisms persisted to the end of the second week, several were very close to going extinct (Fig. S3), allowing us to start the dispersal treatment. Overall, dispersal greatly promoted the persistence of exploited mutualistic communities, with all dispersal-allowed communities surviving through the entire experiment (Fig. 2A). In contrast, 80% of the exploited communities collapsed without dispersal (Fig. 2A). Moreover, dispersal also prevented species loss in the mutualist-only communities. With dispersal allowed, no species went extinct in the mutualist-only communities, whereas there were only 25% and 45% of isolated, mutualist-only communities that had no species loss of AdeOPs and LysOPs, respectively (Fig. 2A). Alpha diversity measured as Shannon index (*q* = 1) of dispersal-allowed communities was significantly higher than the isolated communities in both exploited and mutualist-only systems (MCMCglmm, posterior mean = 2.7906; 95% CI = [2.4831, 3.1516]; ESS = 2100; *P*_MCMC_ < .001) (Fig. 2B and C). Aside from the rescue effect of dispersal, we found that alpha diversities in exploited communities were significantly lower than in mutualist-only communities without dispersal, indicating that cheaters negatively affected the alpha diversity of mutualistic communities (MCMCglmm, Posterior mean = −0.3916; 95% CI = [−0.5503, −0.2407]; ESS = 2100; *P*_MCMC_ < .001; Fig. 2B and C).

**Figure 3:**
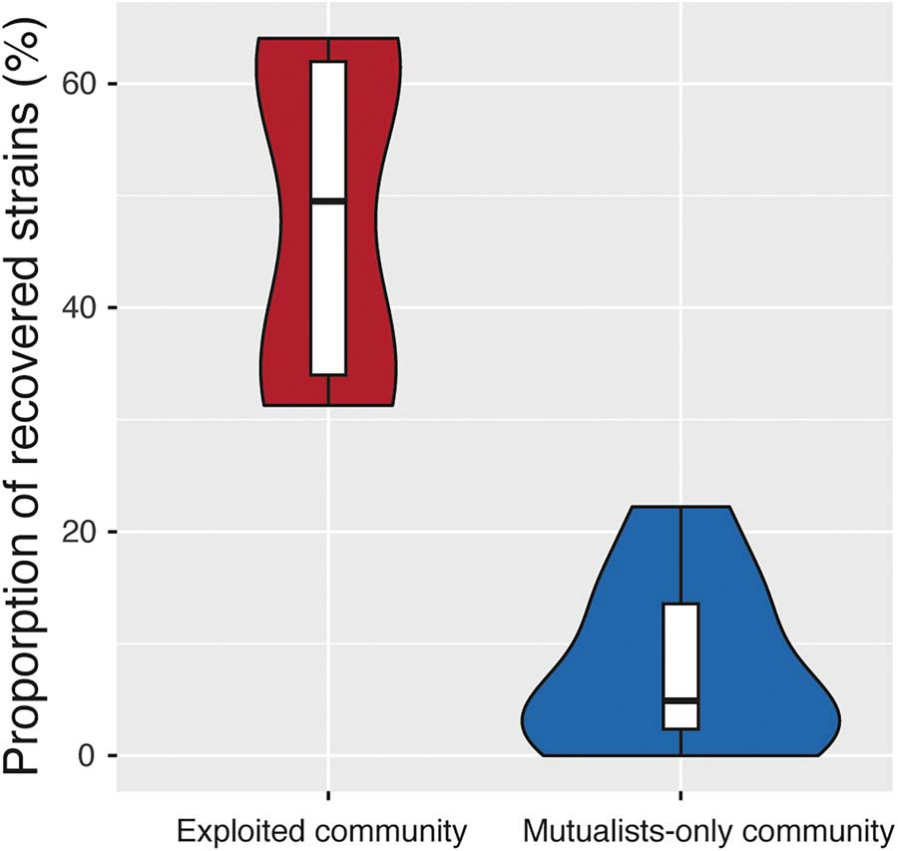
The proportion of strains recovered after dispersal (strains reaching high density after introduction) was higher in exploited communities than in mutualist-only communities, indicating that the rescue effect of dispersal was significantly stronger in exploited communities.

We examined the potential differences in the rescue effect of dispersal by measuring the abundance of each strain after dispersal. Our results showed that the proportion of strains recovered in exploited communities after dispersal was nine times higher than in mutualist-only communities (GLMM, Estimate ± SE = 0.484 ± 0.04, *z* = 12.4, *P* = <.001; Fig. 3).

### Dispersal reduces beta diversity and stabilizes mutualistic community structure

**Figure 4:**
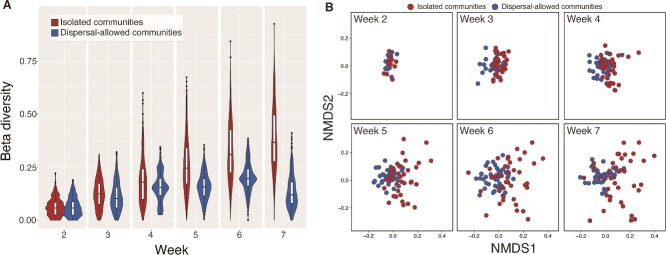
Beta diversity of mutualist-only communities across different weeks. (A) Beta diversity remained stable over time in dispersal-allowed communities, whereas it increased significantly in isolated communities. (B) NMDS plots based on beta diversity show that dispersal-allowed communities consistently clustered together over time, whereas isolated communities became more scattered, indicating that dispersal stabilized community structure. Points represent individual communities across weeks.

Beta diversity among the mutualistic communities stayed at the same level throughout the experiment when dispersal was allowed (GLMM, Estimate ± SE = −0.0015 ± 0.001, *z* = −1.2, *P* = .222; Fig. 4A). In contrast, beta diversity among the isolated communities increased significantly over time (GLMM, Estimate ± SE = 0.05 ± 0.001, *z* = 73.3, *P* < .001; Fig. 4A). Beta diversity in all communities was consistent with null expectations based on randomly assembled communities with the same number of strains, suggesting that the elevated beta diversity in isolated communities was due to random species loss at local communities (Mann–Whitney test, *P* = .645). Moreover, the dispersal-allowed communities consistently clustered together during the 7 weeks of the experiment, whereas the isolated communities overdispersed greatly based on the NMDS plot, especially in the last 2 weeks of the experiment (Fig. 4B). These results indicated that dispersal regionally stabilized the structure of mutualistic communities.

## Discussion

Dispersal has the potential to promote mutualism persistence and stability, but it had yet to be tested experimentally. The results from our experiment highlight the importance of dispersal in facilitating the persistence of multispecies mutualistic communities, especially when facing exploitation. We found that dispersal significantly increased the persistence of exploited communities by 80% (communities with at least one species persisting). Moreover, we found that dispersal increased local alpha diversity, reduced beta diversity among communities, and stabilized community structure at the regional scale. Taken together, our study provides new insights for understanding the persistence of multispecies mutualistic communities and has important implications for biodiversity conservation in the current era of human-induced global change.

### Ecological dynamics of yeast mutualistic communities

The negative effect of a cheater on mutualisms was evident even before we allowed dispersal among communities. We found that the abundance of all strains, but especially of adenine mutualists, was significantly lower in the communities exploited by the lysine cheater than in the mutualist-only communities. This is unsurprising as the lysine cheater consumed lysine without any contribution, thereby largely increasing the intensity of competition for lysine among the adenine mutualists and between the cheater and adenine mutualists, eventually reducing the abundance of adenine mutualists. Additionally, we found that the presence of the cheater reduced alpha diversity in all communities, even when dispersal was allowed. Previous experiments using the same system also showed that the lysine cheater can be a strong competitor and lead to the breakdown of yeast mutualism communities, potentially because of the dynamics of adenine and lysine availability in the system [4]. Hence, this system represents an extreme case of a cheater who is a strong competitor for the mutualistic resource.

Our experiment also shows an important role of stochasticity in determining community composition. Although several strains started to show very low abundance after 2 weeks, potentially due to competition, there was no correlation between individual strains (species) and their abundance when examining the same mutualistic type. This result suggests random species loss in the local communities, indicating that there is not a specific strong competitor dominating every community. This could be because the four species in each mutualist type overlap in niche requirements and compete almost equally for common resources. Consequently, the coexistence of strains is not locally stable, and strain loss is influenced by stochastic processes [18]. In a community where species are equivalent because they have similar niche requirements, replacement of individuals that died could occur randomly, but species at high densities would have a higher change of occupying this empty space [47]. In the absence of speciation and migration from a reginal pool, these communities would eventually become species-poor, as we see in our isolated communities. Another possible explanation is that the interspecific competition is stronger than the intraspecific competition among the strains. In this scenario, strains with higher initial abundance outcompete and exclude the others [18]. However, all mutualistic strains had the same abundance at the beginning of our experiment, leading to the random species loss we observed after 2 weeks. It is likely that stochasticity and competition are both playing a role in the community dynamics we observed.

### Dispersal promotes local diversity, community persistence, and stability

The effects of dispersal on local biodiversity and community persistence have been well studied both theoretically and empirically [48–51], and we provide empirical evidence for dispersal increasing local diversity in mutualistic communities. Dispersal allows species to colonize new communities, creating source–sink dynamics among local communities and promoting species coexistence at the local and regional scales [18]. In our study, the directional dispersal of strains from more favorable communities to less favorable communities increased their population size, largely preventing species loss and thus facilitating the persistence of mutualistic communities. Indeed, our results showed that dispersal significantly prevented species loss in both exploited and mutualist-only communities compared to those isolated communities (Fig. 2). In addition, we found that the rescue effect of dispersal was particularly pronounced in the exploited communities, where all mutualistic communities survived, whereas only 25% communities persisted to the end without dispersal (Fig. 2).

Our results showed that the rescue effect of dispersal on strains varies between exploited and mutualist-only communities, being significantly higher in the former (Fig. 3). This could be because the cheater was competitively superior to all adenine mutualists and became dominant in most of the communities, lowering the population density of all mutualists and thus enlarging the rescue effect of dispersal. The effect of the cheater in the system is mainly driven by changes in resource dynamics; the cheater is better able to survive lysine starvation at the onset of the mutualism, and potentially reduces the amount of lysine available to the mutualists over time^4^. Thus, the change in resource dynamics driven by the cheater likely altered the competitive landscape, and mutualists in the exploited communities could have evolved to be more competitive than those in the communities without the cheater. Those “evolved” strains would have competitive advantages and, therefore, perform better after colonizing the new communities. Alternatively, the presence of a cheater and its effect on resource dynamics could have increased the selective strength among strains, resulting in mutualists evolving local immunity to cheating or generalized trait matching in response to the mutualistic community [52]. These mutualists would then be more likely to successfully colonize a new patch, leading to local mutualism persistence. Indeed, theoretical models of meta-community dynamics in coevolved mutualisms found that increased trait matching between mutualists under strong coevolutionary selection led to reduced local extinctions [15]. Future investigation of the potential evolution of traits associated with meta-community dynamics could help us understand the mechanisms involved in the rescue effect of dispersal.

At the regional scale, our results revealed a significant stabilizing effect of dispersal on mutualistic communities. We observed a clear trend of increasing beta diversity in isolated communities over time due to the random extinction of species in local communities that led to isolated communities greatly varying in community composition. However, in communities where dispersal was allowed, beta diversity remained largely stable throughout the experiment period. Taken together, our results highlighted the effect of dispersal-induced stability on the yeast mutualistic communities [16, 53, 54]. Aside from the effect of dispersal-induced stability, dispersal can also synchronize population dynamics across different communities, thereby increasing the risk of global extinction [16, 55–57]. However, our study did not observe such synchronization in yeast mutualistic communities as the population dynamics of yeast strains varied greatly at the regional scale. This may be due to the dispersal rate in our study not being high enough to synchronize all populations within the mutualistic communities [16].

Even through the directed dispersal applied in our study was selective, it reflects the rescue effect observed in meta-population and meta-community dynamics, where individuals from high-density source populations recolonize sink habitats [39]. Therefore, the directed dispersal can provide a rescuing effect to threatened or impoverished communities and act as an effective conservation approach in face of global change. This rescue effect could be due to a demographic effect by increasing the population size and reducing the stochastic effect or a genetic effect by increasing the genetic diversity and increasing evolutionary potential or restricting inbreeding. Both demographic and genetic rescues have the potential to reduce population declines, but genetic rescue seems to be more effective [37, 38], and, many times, these effects act together. Although we did not specifically tease apart these mechanisms, our results show that the directed dispersal from a population with high density to a declining population had a rescuing effect and resulted in increased alpha diversity. For mutualists that are experiencing declines associated with global change, e.g. due to habitat destruction or displacement by invasive species, directed re-introduction could be an effective way to maintain mutualistic communities [58]. However, we acknowledge that other dispersal models, such as random dispersal, might offer additional insights into the persistence of community in disturbed habitats. For example, random dispersal, where individuals disperse independently of population densities or other traits could serve as an intermediate scenario between complete isolation and directed dispersal. Future studies could explore the effects of random versus directed dispersal on the persistence and stability of mutualisms.

## Conclusions

Understanding the persistence of multispecies mutualism, especially exploited mutualism, is one of the most pressing concerns in biodiversity and conservation. By applying an experimental ecological approach with yeasts, we create a synthetic exploited mutualistic system to examine the effect of dispersal on the persistence of multispecies mutualisms. Our results echo the previous findings emphasizing the rescue effect of dispersal on biodiversity across spatial scales. We found that dispersal increases local diversity by preventing random extinction and stabilizes the community structure of the mutualistic yeast system. Taken together, our results highlight the essential role of dispersal in maintaining multispecies mutualisms, especially when facing exploitation. Additionally, our results provide experimental evidence that meta-community dynamics can facilitate persistence of mutualisms exploited by cheater that have a negative effect on the mutualism, thus contributing to our understanding of how mutualisms persist despite exploitation. Future research examining the combined effects of dispersal, interspecific competition, and the evolution among mutualists will provide new insights for understanding the responses and persistence of mutualisms.

## Supplementary Material

Yeast_meta_MS_Supplementary_information_ISME_Final_wraf003

## Data Availability

All data from this study are present in the Supplementary Materials. R scripts for statistical analyses are available on GitHub at https://github.com/mayracvidal1/yeast_meta-community
