# Supplementary material for: Dispersal promotes stability and persistence of exploited yeast mutualisms
Source: ISME J. 2025 Jan 9;19(1):wraf003. doi: 10.1093/ismejo/wraf003 (PMC11778857; doi:10.1093/ismejo/wraf003)
Supplement: Yeast_meta_MS_Supplementary_information_ISME_Final_wraf003 [file yeast_meta_ms_supplementary_information_isme_final_wraf003.pdf]

Supplementary Materials for

Dispersal promotes stability and persistence of exploited yeast mutualisms

Cong Liu[1,2*] and Mayra C. Vidal[1*]

1. Biology Department, University of Massachusetts Boston, Boston, MA 02125.
2. Museum of Comparative Zoology, Harvard University, Cambridge, MA 02138.

Total number of pages of Supporting Information: 9 (including cover page)

Number of Tables in Supporting Information: 1

Number of Figures in Supporting Information: 3

Table S1: Genotypes of yeast strains used in our experiments.

Figure S1: Agar plate scoring protocol.

Figure S2: Population density dynamics of strains in each community over time. (a) Mutualist-only plate 1; (b) Mutualist-only plate 2; (c) Exploited community plate 1; (d) Exploited community plate 2. Each subplot represents the results for an individual deep-well plate, with the first half of communities as dispersal-allowed and the second half as isolated. Within the same treatment (column), each row represents an individual community (with community ID mapping the position in the deep-well plate), with the dynamics of AdeOP and LysOP strains shown in separate columns. Asterisks (*) indicate dispersal events for each strain.

Figure S3: Proportion of strains with different abundance in each week. (a) mutualists-only, isolated communities; (b) mutualists-only, dispersal-allowed communities; (c) exploited, isolated communities, (d) exploited, dispersal-allowed communities.

Material and Methods

a. Yeast system

Table S1: Genotypes of yeast strains used in our experiments.

**Strain ID**	**Genotype**	**Positive selection plate**
RY1039<br>(LysOP strain 1)	MAT**a** ste3Δ::kanMX4 ade8Δ0 his3Δ1 leu2Δ0 lys21^{OP}	SD-Lys-Ura-Trp
RY1057<br>(AdeOP strain 1)	MAT**a** ste3Δ::kanMX4 lys2Δ0 his3Δ1 leu2Δ0 ade4^{OP}	SD-Ade-Ura-Trp
RY1063<br>(AdeOP strain 2)	MAT**a** ste3Δ::kanMX4 lys2Δ0 his3Δ1 ura3Δ0 ade4^{OP}	SD-Ade-Leu-Trp
RY1069<br>(AdeOP strain 3)	MAT**a** ste3Δ::kanMX4 lys2Δ0 leu2Δ0 ura3Δ0 ade4^{OP}	SD-Ade-His-Trp
RY1081<br>(AdeOP strain 4)	MAT**a** ste3Δ::kanMX4 lys2Δ0 leu2Δ0 ura3Δ0 trp1Δ63::Hyg^{BR} ade4^{OP}	SD-Ade-His+HygB
RY1084<br>(LysOP strain 2)	MAT**a** ste3Δ::kanMX4 ade8Δ0 his3Δ1 ura3Δ0 trp1Δ63::Hyg^{BR} lys21^{OP}	SD-Lys-Leu
RY1086<br>(LysOP strain 3)	MAT**a** ste3Δ::kanMX4 ade8Δ0 his3Δ1 leu2Δ0 trp1Δ63::Hyg^{BR} lys21^{OP}	SD-Lys-Ura+HygB
RY1088<br>(LysOP strain 4)	MAT**a** ste3Δ::kanMX4 ade8Δ0 leu2Δ0 ura3Δ0 trp1Δ63::Hyg^{BR} lys21^{OP}	SD-Lys-His+HygB
RY1093<br>(Lys cheater)	MAT**a** ste3Δ::kanMX4 lys2Δ0 his3Δ1 ura3Δ0 trp1Δ63::Hyg^{BR} ade4^{WT}	SD-Ade-Leu+HygB

b. Agar plate scoring

The abundance of the strains was scored to the following five levels.

1. **Strong**
   a. Scored as 1
   b. A perfect circle (or circle-ish) with a smooth border (see blue circle).

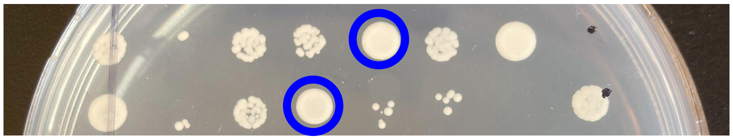

2. **Weak**
   a. Scored as 0.7
   b. It looks like a filled-in circle with a rough border (see purple circles)
3. **Very weak**
   a. Scored as 0.3
   b. Clearly defined colonies can be seen (see green circles)
4. **Micro**
   a. Scored as 0.1
   b. Only very small colonies can be observed (see orange circles)
5. **Extinct**
   a. Scored as 0
   b. No spot on the plate

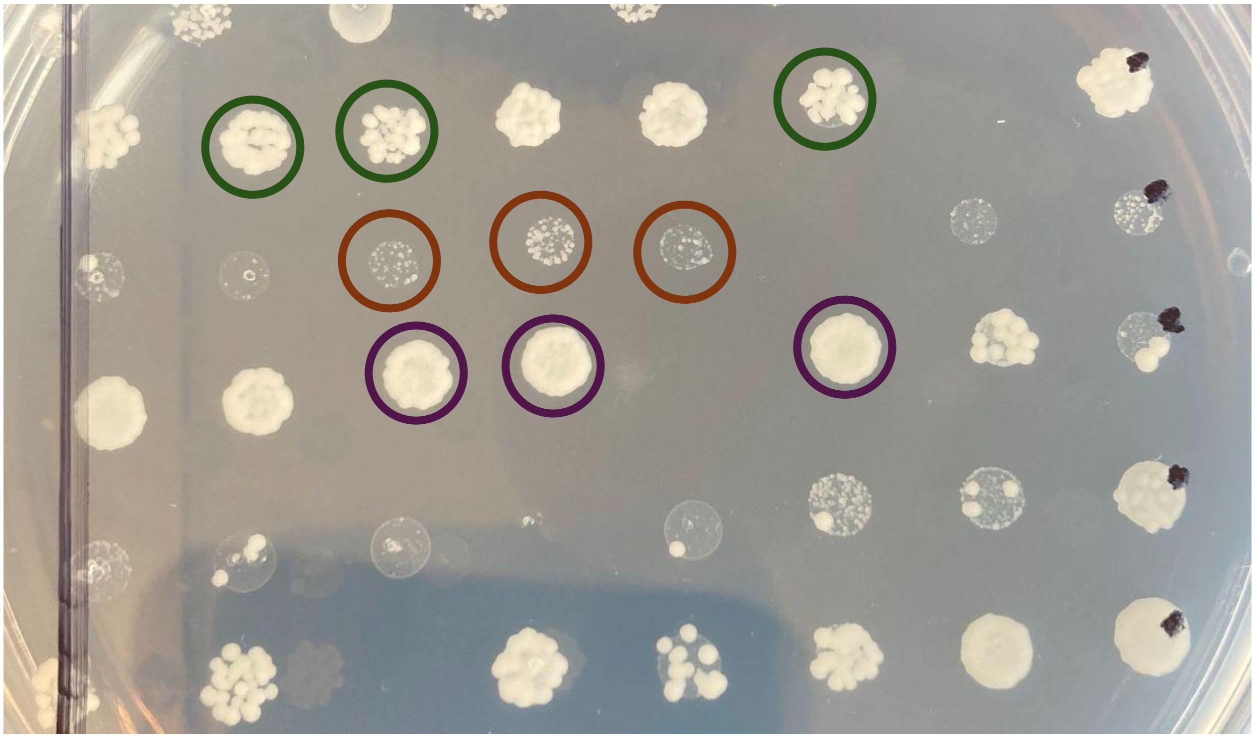

To confirm that these qualitative estimates corresponded to actual population density differences in the liquid media, we quantitatively plated three communities that had all nine strains still present but at varying scoring levels. We found that for both AdeOPs and LysOPs, the CFU/ml varied by scoring level with a 10- to 15-fold increase in density per level comparison (ANOVA with score levels and strain ID as predictors, AdeOPs: $F_{3,15}=16.72$, $P<0.0001$, LysOPs: $F_{3,15}=412.15$, $P<0.0001$; **Fig. S1**). On average, strains scored as 'strong' had 2,414,583 CFU/ml, 'weak' had 151,250 CFU/ml, 'very weak' 14,062 CFU/ml, 'micro' 1,042 CFU/ml, and we did not obtain any colony growth from strains scored as 'gone'.

Quantification of colony-forming units (CFU/ml log transformed) from scoring protocol:

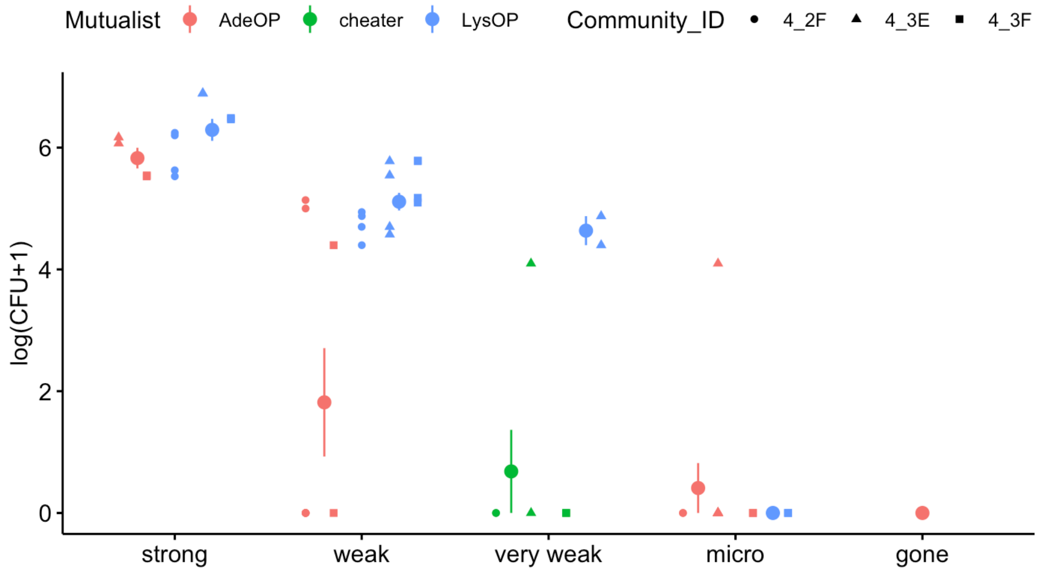

Figure S1. Agar plate scoring protocol.

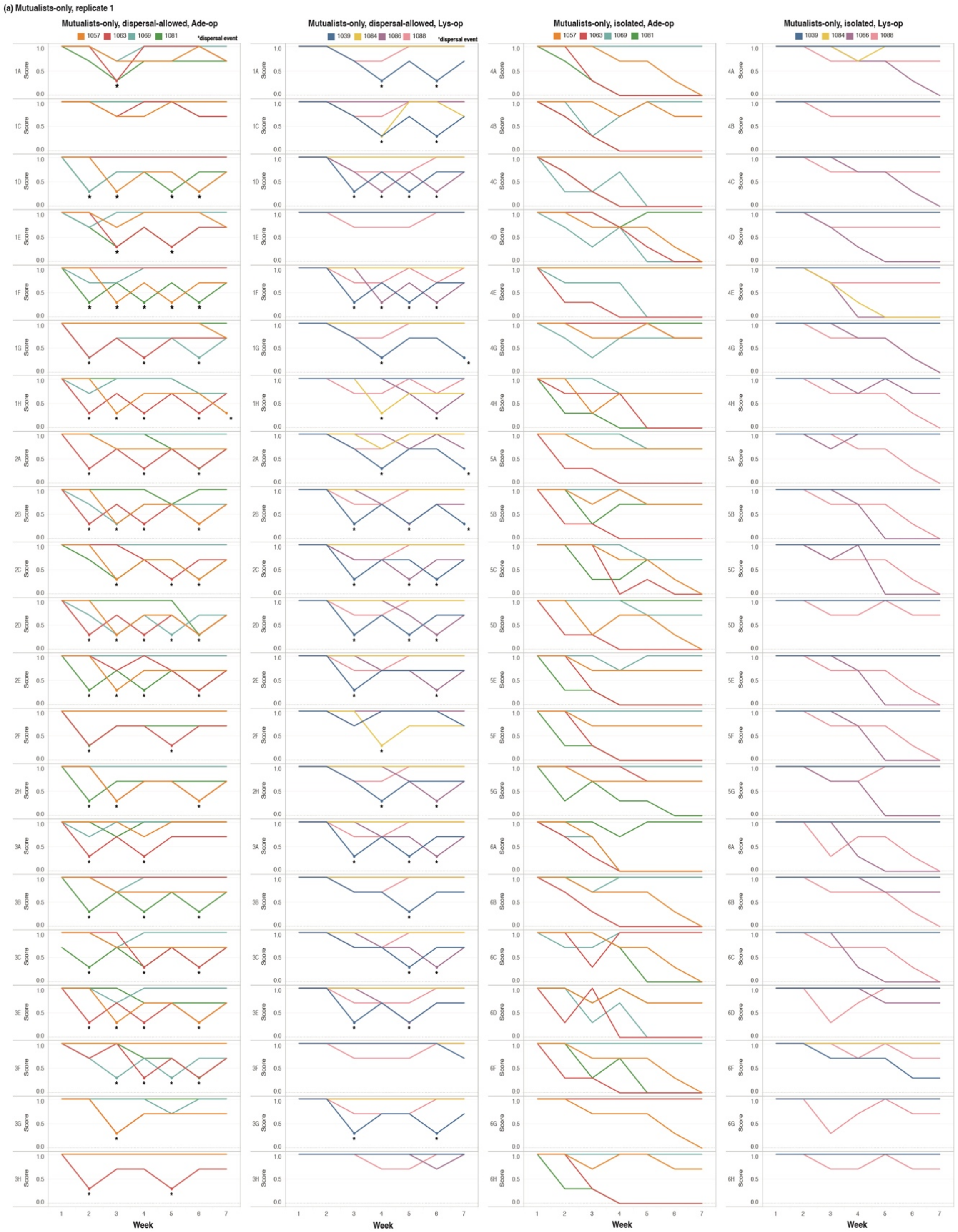

Figure S2. Population density dynamics of strains in each community over time. (a) Mutualist-only plate 1**; (b)** Mutualist-only plate 2**; (c)** Exploited community plate 1**; (d)** Exploited community plate 2. Each subplot represents the results for an individual deep-well plate, with the first half of communities as dispersal-allowed and the second half as isolated. Within the same treatment (column), each row represents an individual community (with community ID mapping the position in the deep-well plate), with the dynamics of AdeOP and LysOP strains shown in separate columns. Asterisks (*) indicate dispersal events for each strain.

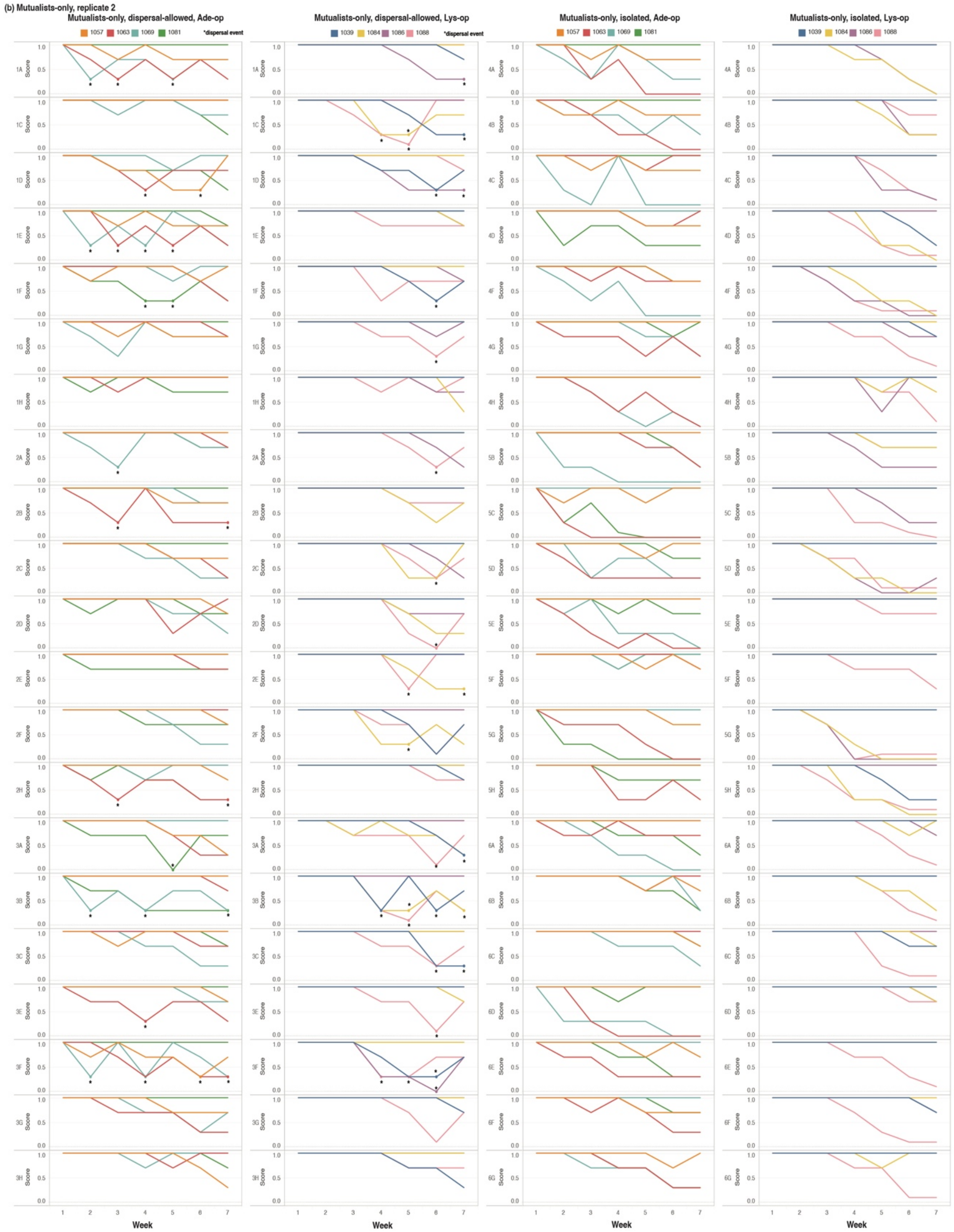

Figure S2 (continued). Population density dynamics of strains in each community over time. (a) Mutualist-only plate 1**; (b)** Mutualist-only plate 2**; (c)** Exploited community plate 1**; (d)** Exploited community plate 2. Each subplot represents the results for an individual deep-well plate, with the first half of communities as dispersal-allowed and the second half as isolated. Within the same treatment (column), each row represents an individual community (with community ID mapping the position in the deep-well plate), with the dynamics of AdeOP and LysOP strains shown in separate columns. Asterisks (*) indicate dispersal events for each strain.

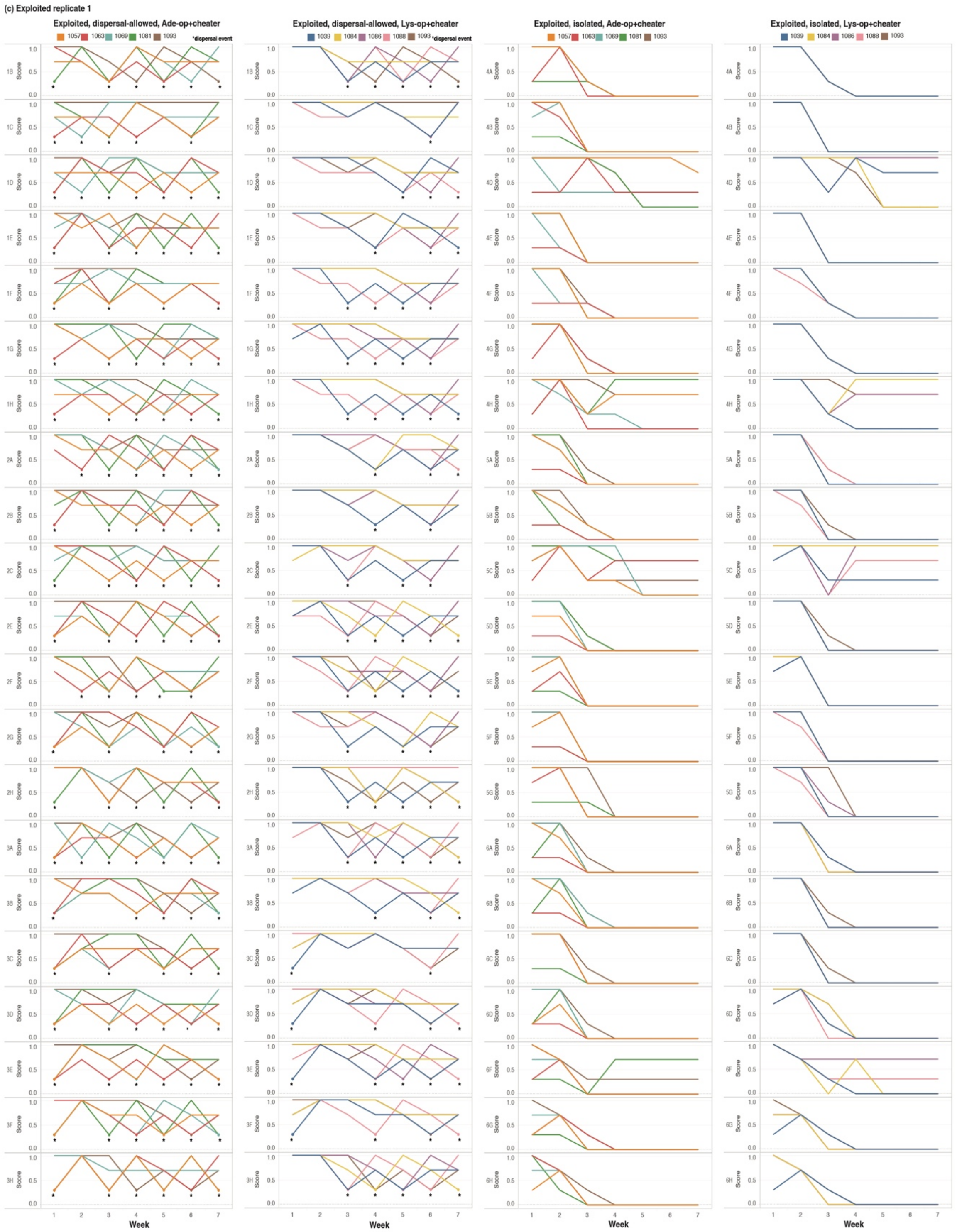

Figure S2 (continued). Population density dynamics of strains in each community over time. (a) Mutualist-only plate 1**; (b)** Mutualist-only plate 2**; (c)** Exploited community plate 1**; (d)** Exploited community plate 2. Each subplot represents the results for an individual deep-well plate, with the first half of communities as dispersal-allowed and the second half as isolated. Within the same treatment (column), each row represents an individual community (with community ID mapping the position in the deep-well plate), with the dynamics of AdeOP and LysOP strains shown in separate columns. Asterisks (*) indicate dispersal events for each strain.

(d) Exploited replicate 2

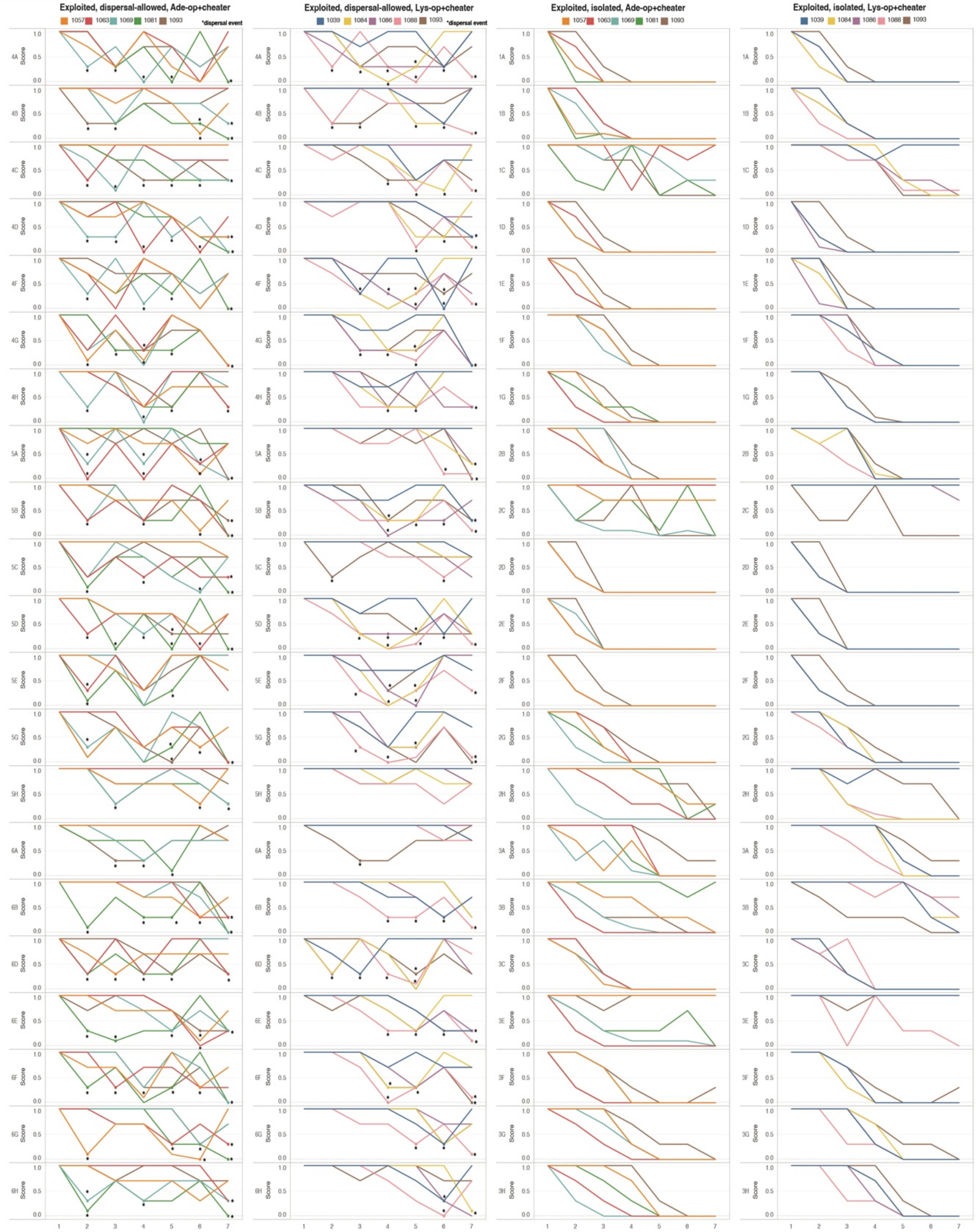

Figure S2 (continued). Population density dynamics of strains in each community over time. (a) Mutualist-only plate 1**; (b)** Mutualist-only plate 2**; (c)** Exploited community plate 1**; (d)** Exploited community plate 2. Each subplot represents the results for an individual deep-well plate, with the first half of communities as dispersal-allowed and the second half as isolated. Within the same treatment (column), each row represents an individual community (with community ID mapping the position in the deep-well plate), with the dynamics of AdeOP and LysOP strains shown in separate columns. Asterisks (*) indicate dispersal events for each strain.

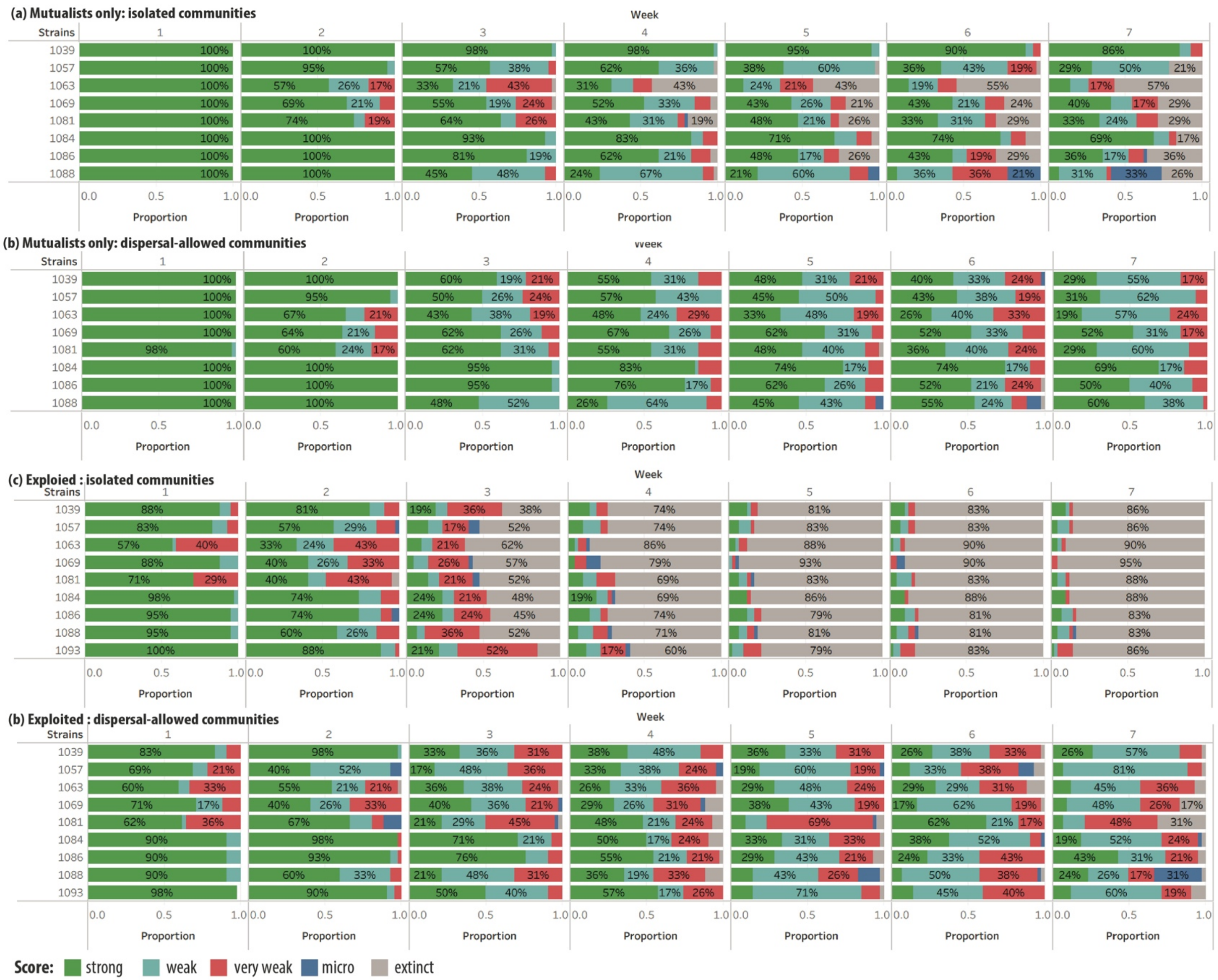

Figure S3. Proportion of strains with different abundance in each week. **(a)** mutualists-only, isolated communities; **(b)** mutualists-only, dispersal-allowed communities; **(c)** exploited, isolated communities, **(d)** exploited, dispersal-allowed communities.
